# Independent Predicting Factors for Subcutaneous Emphysema Associated with Robotic-Assisted Laparoscopic Radical Prostatectomy: A Retrospective Single-Center Study

**DOI:** 10.3390/jcm10132985

**Published:** 2021-07-04

**Authors:** Waichi Yamamoto, Tasuku Nishihara, Taisuke Hamada, Mikiko Takeuchi, Hideyuki Nandate, Sakiko Kitamura, Yasushi Takasaki, Toshihiro Yorozuya

**Affiliations:** Department of Anesthesia and Perioperative Medicine, Ehime University Graduate School of Medicine, Toon 791-0295, Ehime, Japan; 3sk5sj@bma.biglobe.ne.jp (W.Y.); ntasukuk@yahoo.co.jp (T.N.); t.hamada0426@gmail.com (T.H.); fukuanko.5.5.5@gmail.com (M.T.); nandate@hotmail.co.jp (H.N.); sacco926@hotmail.com (S.K.); yorozuya@m.ehime-u.ac.jp (T.Y.)

**Keywords:** subcutaneous emphysema, RALP, complication

## Abstract

Objectives: Subcutaneous emphysema (SCE) is a complication associated with laparoscopic surgery. Severe SCE complicated by excessive hypercarbia may afford detrimental effects in surgical patients with cardiac dysfunction. Robotic-assisted laparoscopic radical prostatectomy (RALP) has several predisposing factors that contribute to SCE. The main purpose of our single-center retrospective study was to determine the preoperative and intraoperative predicting factors for SCE associated with RALP and to determine the actual incidence of SCE. Methods: In total, 229 adult male patients underwent standardized RALP for prostate cancer over the period of 1 May 2016 to 31 October 2018 at the Ehime University Hospital. We reviewed electronic clinical records for individual characteristics including age, body weight, height, coexisting disorders, preoperative ASA physical status, and the length of postoperative hospital stay. We also reviewed surgical and anesthetic records for the operation time, anesthetic method, and the partial pressure of end-tidal CO_2_ (PetCO_2_) during RALP. To determine the presence of SCE, we examined supine chest X-rays obtained after the completion of surgery. Results: We found 55 cases (24.0%) of SCE. Multiple logistic regression analysis showed that a BMI < 25 kg/m^2^ (OR: 3.0, 95% CI: 1.25–7.26) and a maximum value of PetCO_2_ of 46 mmHg or greater (OR: 23.3, 95% CI: 8.22–66.1) were independent predicting factors for SCE. Conclusion: These two predicting factors may be helpful to recognize the occurrence of SCE. Anesthesiologists should protect against SCE progression with the earlier detection of SCE for safe anesthetic management in patients undergoing RALP.

## 1. Introduction

Subcutaneous emphysema (SCE) is a complication associated with laparoscopic surgery and has a reported prevalence below 3.0% [1,2]. However, a previous study on laparoscopic cholecystectomy used computed tomography as a diagnostic tool for SCE, revealing that 56% of patients had the presence of SCE within 24 h of surgery [3]. Recently, robotic-assisted surgery has increased in popularity worldwide for laparoscopic or thoracoscopic surgical procedures [4]. However, exact data for the incidence of SCE are lacking for robotic-assisted surgeries. A robotic-assisted laparoscopic radical prostatectomy (RALP) has several predisposing factors for SCE, such as prolonged surgery duration and multiple surgical ports [5]. Therefore, SCE incidence in RALP may be much higher than previously reported incidences of SCE in other laparoscopic surgeries; however, the actual incidence of SCE in RALP remains unknown.

Common clinical derangements of SCE are hypercarbia and subsequent acidosis, the severity of which depends on the extent of the SCE area [1,6]. Initial treatment for hypercarbia involves increasing pulmonary ventilation during the intraoperative period. However, it may be difficult to set pulmonary ventilation beyond the level that is enough to normalize PaCO_2_, especially in the case of RALP, because chest compliance is limited by a combination of a steep Trendelenburg position and increased intraabdominal pressure caused by CO_2_ insufflation [7,8,9]. If serious cases of SCE occur during RALP, hypercarbia and acidosis may be persistent with insufficient treatment. This may subsequently cause circulatory and metabolic disturbances, resulting in a decompensated, unstable state, especially in elderly patients with the coexistence of cardiac, pulmonary, or renal disorders [10].

For these situations, we should analyze the anesthetic management strategy for patients undergoing RALP to recognize the early occurrence of SCE and prevent the serious progression of SCE. The purpose of this retrospective study was to determine the preoperative and intraoperative predicting factors associated with SCE occurrence for safe anesthetic management and to also identify incidences of SCE during RALP.

## 2. Material and Methods

This retrospective study was approved by the Ethical committee of Ehime University Hospital, Ehime, Japan. The ethical committee waived the requirement for informed consent. Instead, we announced the information regarding this research and the use of the related data on our department website.

We enrolled male patients more than 18 years of age who consecutively underwent RALP to treat prostate cancer during the period of 1 May 2016 to 31 October 2018 at Ehime University Hospital. We recruited 258 male patients from the American Society of Anesthesiologists (ASA) who were of physical status 1 to 3 and were undergoing RALP during the study period. We reviewed electronic clinical records for age, body weight, height, coexisting disorders, smoking history, preoperative ASA physical status, and the length of postoperative hospital stay. We also reviewed surgical and anesthesia records for operation time, anesthetic method, and the partial pressure of end-tidal CO_2_ (PetCO_2_) during RALP. In addition, we examined supine chest X-rays obtained within approximately 10 min after the end of surgery and that were taken in the operation room. When SCE was an extended cephalad over a diaphragmatic border on the postoperative chest X-ray, we confirmed the obvious presence of SCE. Patients were excluded when their chest X-rays findings were inconclusive for the presence of SCE. The choice of the general anesthetic method depended on the attending anesthesiologist. While most patients were maintained with a combination of inhalational anesthetics such as sevoflurane or desflurane with a continuous infusion of remifentanil, other (fewer) patients were maintained with total intravenous anesthesia with propofol and remifentanil. Nitrous oxide gas was not supplemented in any anesthetic method. Rocuronium was administered for the muscle relaxation required for laparoscopic surgery. In addition to standard monitoring tools such as ECG, pulse oximetry, and PetCO_2_, a 22-ga cannula was inserted in the radial artery to directly monitor the arterial pressure. The initial ventilation was set at a level to maintain a PetCO_2_ below 40 mmHg. However, if the PetCO_2_ exceeded 40 mmHg during pneumoperitoneum with CO_2_ insufflation, the minute ventilation volume was increased to keep peak inspiratory pressure less than 35 cmH_2_O, as decided by the attending anesthesiologist. We were unable to determine via anesthesia records which patients had increased minute ventilation, but we were able to record the maximum PetCO_2_ during RALP. Following the induction of general anesthesia, one camera port was inserted at the umbilical region and other four surgical ports were inserted at the bilateral upper abdominal region. Intraabdominal pressure with CO_2_ insufflation was maintained below 12 mmHg throughout the RALP. Patients were placed in the steep Trendelenburg position of 25 degrees, which is the standard for RALP under the DaVinci Si surgical system.

The presence of obvious SCE during RALP was a main outcome of this study. Patients were allocated to SCE (+) or SCE (−) groups according to the findings of their postoperative chest X-rays for the confirmation of obvious SCE. All statistical analyses were performed with EZR (Saitama Medical Center, Jichi Medical University, Saitama, Japan), which is a graphical user interface for R (The R Foundation for Statistical Computing, Vienna, Austria). Student’s t test was used to analyze numerical data, and Fisher’s exact test was used for categorical data to compare the two SCE groups. On the basis of the previously reported clinical usefulness regarding laparoscopic surgery and this univariate comparison, we created the four following binomial categories: an age of more than 70 years, a body mass index (BMI) < 25 kg/m^2^, an operation time longer than 200 min, and a maximum PetCO_2_ of 46 mmHg or greater. Multiple logistic regression analysis was employed for these categorical variables thus identifying independent factors predicting SCE incidence during the RALP. *p* < 0.05 was considered statistically significant.

## 3. Results

**Figure 1:**
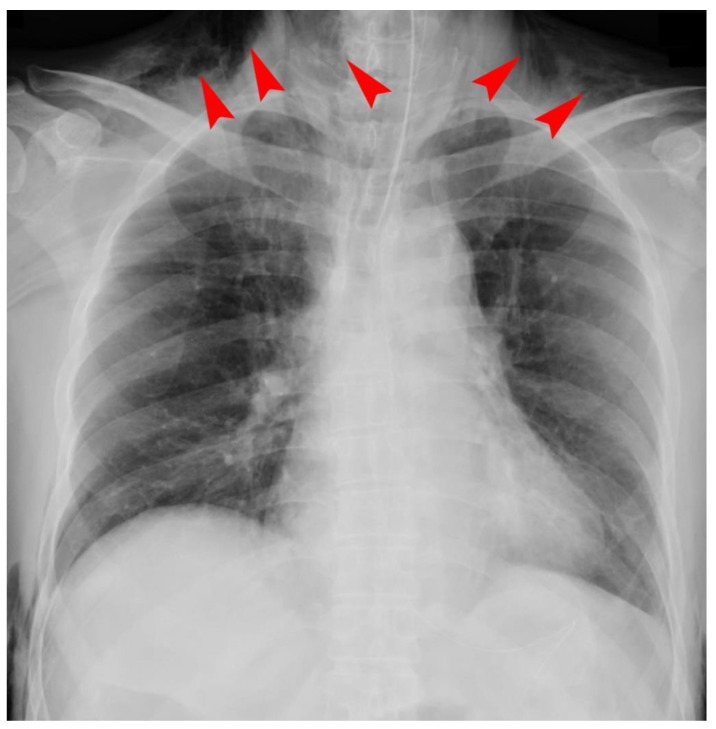
A chest X-ray of a serious case of subcutaneous emphysema. Subcutaneous emphysema (SCE) massively extended as far as the neck region in 12 serious cases among 55 SCE (+) cases. Arrowheads indicate SCE in the neck region.

We excluded 29 patients because their chest X-ray findings were inconclusive regarding whether SCE was present or not after the examination of two researchers. We included 229 patients who consecutively underwent RALP in the final analysis. Among them, 209 patients received inhalational anesthesia, and 20 patients received total intravenous anesthesia. We found 55 cases (24.0%) of SCE and no cases of pneumothorax or pneumomediastinum after examining the postoperative chest X-rays. Among the 55 cases of SCE, 12 serious cases had patients with SCE massively extending as far as the neck region (Figure 1). Although the primary clinical sign in such severe cases was sustained hypercarbia exceeding 45 mmHg in PetCO_2_, we neither recognized any postoperative problems for upper airway patency nor any difficulty in breathing because of the rapid resolution of the hypercarbia after terminating pneumoperitoneum with CO_2_ insufflation. With the exception of one patient with chronic renal failure who needed to receive hemodialysis in the SCE (−) group, no significant difference was noted in any coexisting disorders such as hypertension, diabetes mellitus, hyperlipidemia, smoking history, and abnormal pulmonary function of the obstructive type between the groups (Table 1). In addition, the development of SCE during RALP did not significantly influence the length of postoperative hospitalization (Table 1). The univariate comparison determined that BMI was significantly less than and the maximum PetCO_2_ in SCE (+) group was significantly higher than that of the SCE (−) group (Table 1). There was no significant difference in age or operation time the between groups. Multiple logistic regression analysis determined two independent predicting factors for SCE associated with RALP: BMI < 25 kg/m^2^ (OR: 3.0, 95% CI: 1.25–7.26) and a maximum PetCO_2_ of 46 mmHg or greater (OR: 23.3, 95% CI: 8.22–66.1) (Table 2).

## 4. Discussion

In this retrospective study, we evaluated the incidence of SCE in patients undergoing RALP by examining postoperative chest X-rays to identify several predicting factors SCE associated with RALP. The incidence of SCE in our cohort was approximately 24%, and we determined that a BMI < 25 kg/m^2^ and maximum PetCO_2_ of 46 mmHg or greater were independent predicting factors for SCE associated with RALP.

The incidence of SCE in our patients who were undergoing RALP was much higher than previously reported in laparoscopic surgeries [1]. We cannot simply compare the incidence of SCE between the present study and the previous studies because of several differences in each reported laparoscopic surgery, such as the number of ports, insufflation pressure of CO_2_, operation time, and patient position. However, one previous study used computed tomography as a diagnostic tool and showed that 56% of patients undergoing uncomplicated laparoscopic cholecystectomy had the presence of obvious SCE [3]. The diagnosis of SCE presence may vary depending on the diagnostic modality used. Although chest X-rays and computed tomography are thought to be more sensitive in the detection SCE compared with a simple palpation, they may overestimate SCE because clinically insignificant SCE is likely included in those cases that are diagnosed as SCE. Among the 55 cases with SCE in this study, we confirmed 12 serious cases (21.8%) in whom SCE was massively extended to the neck region on a postoperative chest X-ray. We recommend that a chest X-ray should be taken after RALP to avoid overlooking the presence of serious SCE and to determine the extent of SCE because of the convenience of an operating room chest X-ray. If pneumothorax or pneumomediastinum is suspected from a postoperative chest X-ray, we should subsequently consider performing computed tomography [11,12].

The progression of SCE formation is thought to begin at the insertion site of the surgical ports penetrating the abdominal wall [13]. There are several possible causes related to SCE formation, such as excessive intraabdominal pressure, higher insufflated gas flow, and unexpectedly robust force around the surgical ports, especially when surgeons remotely manipulate robot arms in robotic-assisted laparoscopic surgeries [1]. Furthermore, this manipulation tends to damage more vulnerable structures in the subcutaneous tissue around the surgical ports. Subsequently, insufflated gas easily invades dissected tissue, resulting in the progression of SCE formation. We found that a BMI < 25 kg/m^2^ was an independent predicting factor for SCE, yet the World Health Organization classifies BMI < 25 kg/m^2^ as normal or underweight for Asian populations [14]. A Japanese study investigating the relationship between BMI and the subcutaneous fat area (SFA) at the umbilical level showed that BMI was closely correlated with SFA [15], which suggests that patients with a BMI higher than 25 kg/m^2^ of BMI (overweight or obese) have a substantially thicker layer of subcutaneous fat in their abdomen. The thickness of the subcutaneous fat may be associated with some protective effects against SCE formation caused by several minor injuries. A simple preoperative assessment of a BMI < 25 kg/m^2^ can alert surgeons and anesthesiologists to a higher risk of SCE incidence.

While a PetCO_2_ of 50 mmHg or more has been previously reported as an independent predicting factor for SCE [3], we found a PetCO_2_ threshold of 46 mmHg in our study. We were unable to find any information regarding the ventilation conditions in that previous report [5], but one possible reason for our reduced PetCO_2_ threshold is the adjustment of ventilation by the attending anesthesiologist to resolve hypercapnia. If the ventilation was not adjusted for hypercarbia, perhaps our PetCO_2_ threshold would have been near the previously reported 50 mmHg. Because remarkable alterations in PetCO_2_ are important warning signs for SCE incidence during RALP, the trend of PetCO_2_ should be carefully monitored. If the PetCO_2_ reaches approximately 46 mmHg despite increasing the minute ventilation volume, we should consider SCE development and take preventive measures against the further progression of SCE. We did not identify other well-established predicting factors such as a patient age of more than 65 years, an operation time of longer than 200 min, or 5 or more surgical ports [5], likely because of our limited cohort.

There are several limitations to our retrospective study which warrant discussion. First, we unintentionally excluded 29 patients who accounted for over 10% of the enrolled patients from this study because of unclear chest X-ray findings for SCE. However, this exclusion may be regarded as a type of selection bias. If none of them have suffered from SCE, the odds of SCE incidence were 0.271 (95%CR, 0.211–0.338). The odds ratio to the original data of SCE incidence would then be 0.875 (95%CR, 0.626–1.174). Therefore, we should carefully interpret the result by considering this extreme assumption. Second, as we mentioned earlier, to some extent, this study includes patients with clinically insignificant SCE, which appears to be limited to a small area, even if the SCE has extended beyond the diaphragmatic border. This is because we only diagnosed SCE by examining the postoperative chest X-ray. We did not select patients with clinically significant SCE alone because of the lack of clear definition of clinically significant SCE and an estimated smaller number of possible patient selections. It is likely that the PetCO_2_ threshold to predict SCE would differ if subclinical SCE cases were excluded. Moreover, due to the retrospective nature of our study, the attending anesthesiologist did not always provide similar anesthetic management, including differences in anesthetic, ventilatory settings, and infused fluid volume. This may substantially influence our study but reflects the real-world clinical setting in which this study was based on.

## 5. Conclusions

In conclusion, this retrospective study evaluated the incidence of SCE in male patients undergoing RALP and demonstrated two independent predicting factors for SCE during RALP: a BMI < 25 kg/m^2^ and a maximum PetCO_2_ of 46 mmHg or greater. These two factors may help anesthesiologists in recognizing the early occurrence of SCE and protect patients against the progression of SCE during RALP.

## Figures and Tables

**Table 1:** Demographic data and clinical characteristics of 229 patients. A case of massive subcutaneous emphysema extending to the neck region. Numerical data are presented as mean (SD). Categorical data are presented as number (%). SCE, subcutaneous emphysema; ASA PS, American Society of Anesthesiologists physical status; Obstructive type of pulmonary function is defined by %FEV1.0 of less than 70%. *p*-value < 0.05 is considered significant.

	SCE (−)	SCE (+)	
	(n = 174)	(n = 55)	*p* Value
Age (year)	69 (6.7)	70 (5.9)	0.13
Body mass index (kg/m^2^)	24.2 (2.8)	23.0 (2.3)	0.004
Operation time (min)	185.9 (50.6)	192.6 (52.3)	0.39
Maximun end-tidal CO_2_ (mmHg)	38.4 (3.4)	44.3 (5.8)	<0.001
ASA PS 2 or 3	150 (86.2)	49 (89.1)	0.65
Anesthetic method			0.42
inhalational anesthesia	157 (90.2)	52 (94.5)	
total intravenous anesthesia	17 (9.8)	3 (5.5)	
Hypertension	95 (54.6)	30 (54.5)	1
Diabetes millitus	36 (20.7)	6 (10.9)	0.11
Hyperlipidemia	49 (28.2)	18 (32.7)	0.5
Smoking history (longer than 5 years)	94 (54.0)	29 (52.7)	0.88
Abnormal pulmonary function (obstructive type)	44 (25.3)	16 (29.1)	0.7
Length of postoperative hospital stay (day)	10.8 (3.7)	11.2 (3.0)	0.55

Subcutaneous emphysema (SCE) massively extended as far as the neck region in 12 serious cases among 55 SCE (+) cases. Arrowheads indicate SCE in the neck region.

**Table 2:** Independent predicting factors associated with RALP. BMI, body mass index; OPT, operation time; ETCO_2_, end-tidal CO_2_. *p*-value < 0.05 is considered significant.

	Odds Ratio	95% Confidential Range	*p* Value
Age ≧ 70 (year)	1.6	0.78–3.31	0.2
BMI < 25 (kg/m^2^)	3	1.25–7.26	0.0014
OPT ≧ 200 (min)	1.3	0.61–2.71	0.5
Max ETCO_2_ ≧ 46 (mmHg)	23.3	8.22–66.1	<0.001

## Data Availability

The data presented in this study are available upon request from the corresponding author.
